# Preparing the Perfect Cuttlefish Meal: Complex Prey Handling by Dolphins

**DOI:** 10.1371/journal.pone.0004217

**Published:** 2009-01-21

**Authors:** Julian Finn, Tom Tregenza, Mark Norman

**Affiliations:** 1 Sciences, Museum Victoria, Melbourne, Victoria, Australia; 2 Department of Zoology, La Trobe University, Victoria, Australia; 3 Centre for Ecology and Conservation, School of Biosciences, University of Exeter, Cornwall Campus, Tremough, Penryn, United Kingdom; Georgia State University, United States of America

## Abstract

Dolphins are well known for their complex social and foraging behaviours. Direct underwater observations of wild dolphin feeding behaviour however are rare. At mass spawning aggregations of giant cuttlefish (*Sepia apama*) in the Upper Spencer Gulf in South Australia, a wild female Indo-Pacific bottlenose dolphin (*Tursiops aduncus*) was observed and recorded repeatedly catching, killing and preparing cuttlefish for consumption using a specific and ordered sequence of behaviours. Cuttlefish were herded to a sand substrate, pinned to the seafloor, killed by downward thrust, raised mid-water and beaten by the dolphin with its snout until the ink was released and drained. The deceased cuttlefish was then returned to the seafloor, inverted and forced along the sand substrate in order to strip the thin dorsal layer of skin off the mantle, thus releasing the buoyant calcareous cuttlebone. This stepped behavioural sequence significantly improves prey quality through 1) removal of the ink (with constituent melanin and tyrosine), and 2) the calcareous cuttlebone. Observations of foraging dolphin pods from above-water at this site (including the surfacing of intact clean cuttlebones) suggest that some or all of this prey handling sequence may be used widely by dolphins in the region. Aspects of the unique mass spawning aggregations of giant cuttlefish in this region of South Australia may have contributed to the evolution of this behaviour through both high abundances of spawning and weakened post-spawning cuttlefish in a small area (>10,000 animals on several kilometres of narrow rocky reef), as well as potential long-term and regular visitation by dolphin pods to this site.

## Introduction

Dolphins, particularly those of the genus *Tursiops*, are well documented as having diverse foraging abilities and behaviours. Beyond direct prey capture, these include use of the rostrum to dig into soft-sediment substrates [1], deeper sediment “crater feeding” [2], tool-use in the form of marine sponges worn on the rostrum tip to safely probe in soft sediments [1], [3], use of the tail to “whack” fish [4], tail smacks on the water surface above seagrass beds to flush prey (“kerplunking”) [5], co-operative fish herding [6], stirring up sediment to trap fish [7], chasing individual fish onto exposed sand or mudflats (“beach hunting”) [8] and co-operative generation of a pressure wave of water to similarly wash schools of small fish on to exposed intertidal flats, again followed by dolphin beaching to consume the stranded fish [9].

Such foraging behaviours may be performed individually or in groups. Techniques are potentially learned individually and/or transferred between generations or amongst populations through cultural transmission [10], genetic transmission or a combination of both [11].

To date the majority of feeding observations of dolphins have been made from above water, based on surface or near-surface feeding behaviours visible from boats or shore [1], [12]. Direct underwater observations are less common [2].

We report underwater observations and filming of novel feeding behaviours for wild Indo-Pacific bottlenose dolphin (*Tursiops aduncus*) feeding on giant cuttlefish (*Sepia apama*) in South Australia. Annually from May to August, this cuttlefish species forms mass breeding aggregations on narrow strips of rocky reef in the northern Spencer Gulf, South Australia [13], [14]. Tens of thousands of cuttlefish gather each year in this region on a narrow band of reef several kilometres in length in order to mate and spawn. Due to the semelparous reproduction typical of cephalopods, individual cuttlefish have one breeding season then die [13]. As a consequence, the spawning site contains many weakened post-spawning individual cuttlefish.

Pods of foraging Indo-Pacific bottlenose dolphins gather annually at this site and make regular foraging passes over shallow reefs and sand in groups of up to 15 individuals. During repeat visits to this site, an individual dolphin was observed and filmed using a consistent sequence of prey manipulation behaviours in the capture, killing and consumption of wild cuttlefishes.

## Materials and Methods

In May 2003 and May 2007, dolphin feeding behaviour was observed underwater while SCUBA diving near Whyalla, Spencer Gulf in depths of less than 5 m. High definition video footage was taken of this behaviour using a Sony HD Cam video camera. Observers/camera operators remained static on the substrate while dolphin behaviour was observed and filmed.

## Results

### Study animal

A single female dolphin was observed foraging during daylight hours in both 2003 and 2007. Over the course of both visits, a total of 7 cuttlefish were observed to be handled and consumed (2003: n = 4; 2007: n = 3). Based on body markings (circular scars on the head), the dolphin was recognised as the same individual in both years.

### Behavioural sequence

**Figure 1:**
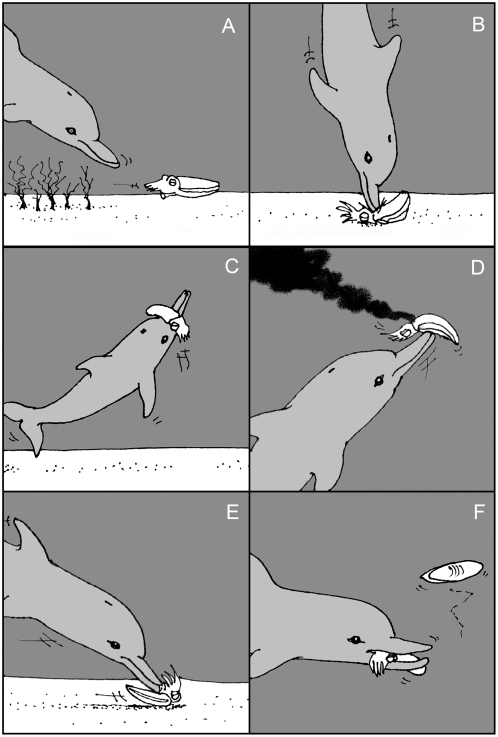
Stages of prey handling of giant cuttlefish (*Sepia apama*) by Indo-Pacific bottlenose dolphin (*Tursiops aduncus*). Prey: (a) flushed from algal cover to open substrate, b) pinned to substrate and killed; (c) lifted towards surface; (d) beaten with snout to release ink; (e) returned to substrate, inverted and forced along the sand to remove skin layer and release cuttlebone, (f) consumed whole.

**Figure 2:**
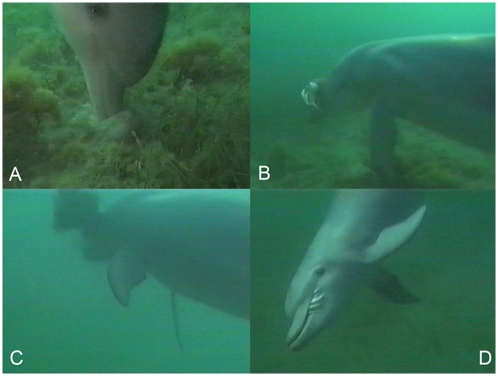
Prey handling of giant cuttlefish (*Sepia apama*) by Indo-Pacific bottlenose dolphin (*Tursiops aduncus*). Prey: (a) pinned to substrate and killed; (b) lifted towards surface; (c) beaten with snout to release ink; (d) consumed whole.

For all cuttlefish taken by this dolphin, a consistent and ordered sequence of prey handling and consumption was observed. Six distinct stages were recognised:

1. *Prey positioning*: Cuttlefish prey were typically hiding amongst dense brown algae. On encountering the cuttlefish, the dolphin flushed the prey away from algal cover into areas of open sand (Fig. 1a).
2. *Prey restraint*: The dolphin then adopted a vertical position in the water column and pinned the prey down against the sand substrate.
3. *Pinned thrust kill*: A rapid downward vertical thrust was effected by the dolphin using a powerful tail beat (Fig. 1b, 2a), accompanied by a whole body twist that broke the cuttlebone and/or cephalic cartilage (with a loud click audible to divers), instantly killing the cuttlefish.
4. *‘Snout beating’ of the corpse*: The corpse was then lifted into the water column on top of the beak (Fig. 1c, 2b) and repeatedly hit with the snout (up to 6 times), until dense clouds of ink were released (Fig. 1d, 2c). Beating continued until ink release diminished.
5. *Removal of intact cuttlebone*: The dead prey was then returned to the sand where it was inverted and the dorsal surface of the cuttlefish body forcibly pushed into and along the sand substrate (Fig. 1e), thus scraping off the thin dorsal skin of the cuttlefish and releasing the cuttlebone, which then floated to the surface.
6. *Ingestion*: The prepared cuttlefish was then consumed whole (Fig. 1f, 2d), or when the head and body were separated during beak beating, only the head was consumed (with attached digestive tract organs).

In addition to our observations, individual bottlenose dolphins feeding at these cuttlefish spawning grounds have been observed by divers in the area to perform the same behavioural sequence (T. Bramley, Whyalla Dive Services, pers. comm.).

### Above-water observations

At least the skin sloughing stage (with resultant release of an intact cuttlebone) appears prevalent at this site as boat-based observers have witnessed clean intact cuttlebones floating to the surface on numerous occasions in the proximity of pods of foraging dolphins (JF pers. obs. 1999; T. Bramley, Whyalla Dive Services, pers. comm.).

## Discussion

The feeding behaviour reported here is specifically adapted to a single prey type and represents impressive behavioural flexibility for a non-primate animal. The key components of this prey-handling sequence are the beating of the corpse mid-water (in order to release the ink) and the scraping of the cuttlefish's dorsal body surface along the sand (in order to release the cuttlebone). Both behaviours dramatically improve prey quality. Cephalopod ink consists primarily of the pigment melanin (found to inhibit gastric secretions, [15]), as well as chemicals considered to impair chemoreception (particularly tyrosinase, [16]). Ink removal would improve both palatability and internal digestive processes. Similarly, sloughing of the dorsal mantle skin is an effective means of releasing the rigid calcareous cuttlebone intact, compared with the more difficult extraction of shattered cuttlebone shards from a masticated cuttlefish.

The prevalence of this behaviour amongst dolphins at the giant cuttlefish spawning site is unknown. Repeated above-water observations of clean cuttlebones bobbing to the surface in association with passing pods of dolphins suggest that some or all of this behavioural sequence is not restricted to a single individual dolphin.

This leaves open the potential for transmission/learning of the foraging behaviour between members of this bottlenose dolphin population. Such transmission has been documented in other animal groups and can be cultural, genetic or a combination of both. Primates are the best-known for cultural transmission of tool use [17], [18]. By contrast, crows bred in captivity possess tool manufacturing abilities in the absence of parental or population influences, indicating a genetic component to this ability [19]. Learning and cultural transmission of behaviours has been reported widely in cetaceans [see 20], with some authors suggesting that a combination of both cultural and genetic transmission can occur [11].

In the current study, the individual dolphin observed preying on cuttlefish was female. Females have been found to play a critical role in transmission of behaviours amongst cetaceans. In referring to female dominance of bottlenose dolphin beach hunting behaviours, Sargeant et al [8] found that “maternal influence appears to be particularly important in *Tursiops* spp” (p. 1408). Bottlenose dolphins from Shark Bay in Western Australia similarly show a female sex bias in protective sponge-use while foraging, while the male offspring of female spongers did not adopt this form of tool use. The origins and potential transmission of complex cuttlefish prey handling behaviours in the Spencer Gulf bottlenose dolphins await further studies.

This behaviour is a dramatic example of how dolphins, with their relatively unspecialised morphology, can utilise behavioural flexibility to tackle prey items that require substantial handling before consumption. The abundance of weakened, dying and dead cuttlefish at the Spencer Gulf mass breeding aggregation [13] provides dolphins with a rich seasonal food supply. The high nutritive value of these cephalopods and the potential longevity of this cuttlefish breeding site may have enabled the evolution of the feeding behaviours reported here. In comparison with the high-speed jet escape of healthy cuttlefish, weakened post-spawning individuals can easily be manipulated, providing the potential for individual learning and/or social transmission of this feeding behavioural sequence to inexperienced or less deft younger dolphins.
